# Toxicological Profile of Umbilical Cord Blood-Derived Small Extracellular Vesicles

**DOI:** 10.3390/membranes11090647

**Published:** 2021-08-24

**Authors:** Silvia C. Rodrigues, Renato M. S. Cardoso, Claudia F. Gomes, Filipe V. Duarte, Patricia C. Freire, Ricardo Neves, Joana Simoes-Correia

**Affiliations:** 1 Exogenus Therapeutics, S.A., Biocant Park, Núcleo 4, Lote 2, 3060-197 Cantanhede, Portugal; silviacouto25@gmail.com (S.C.R.); renatocardoso83@gmail.com (R.M.S.C.); claudiafgomes.94@gmail.com (C.F.G.); filipevalenteduarte@gmail.com (F.V.D.); patricia.freire@exogenus-t.com (P.C.F.); ricardo.neves@uc-biotech.pt (R.N.); 2 Doctoral Programme in Experimental Biology and Biomedicine (PDBEB), Institute for Interdisciplinary Research (IIIUC), Center for Neuroscience and Cell Biology (CNC), University of Coimbra, 3004-531 Coimbra, Portugal; 3 Center for Neurosciences and Cell Biology (CNC), University of Coimbra, 3004-517 Coimbra, Portugal; 4 Institute for Interdisciplinary Research (3Is), University of Coimbra, 3030-789 Coimbra, Portugal

**Keywords:** extracellular vesicles, umbilical cord blood, EV therapeutics, EV toxicity

## Abstract

The development and adoption of cell therapies has been largely limited by difficulties associated with their safety, handling, and storage. Extracellular vesicles (EV) have recently emerged as a likely mediator for the therapeutic effect of cells, offering several advantages over cell therapies. Due to their small size and inability to expand and metastasize, EV are generally considered safer than cell transplantation. Nevertheless, few studies have scrutinized the toxicity profile of EV, particularly after repeated high-dose administration. The present study aimed to evaluate a preparation of small EV obtained from umbilical cord blood mononuclear cells (UCB-MNC-sEV) for its cytotoxicity in different cell lines, as well as its differential accumulation, distribution, and toxicity following repeated intravenous (IV) administrations in a rodent model. In vitro, repeated sEV exposure in concentrations up to 1 × 10^11^ particles/mL had no deleterious impact on the viability or metabolic activity of peripheral blood mononuclear cells, THP-1 monocytes, THP-1-derived macrophages, normal dermal human fibroblasts, or human umbilical vein endothelial cells. DiR-labelled sEV, injected intravenously for four weeks in healthy rats, were detected in clearance organs, particularly the kidneys, spleen, and liver, similarly to control dye. Moreover, repeated administrations for six and twelve weeks of up to 1 × 10^10^ total particles of sEV dye were well-tolerated, with no changes in general haematological cell counts, or kidney and liver toxicity markers. More importantly, unlabelled sEV likewise did not induce significant alterations in cellular and biochemical blood parameters, nor any morphological changes in the heart, kidney, lung, spleen, or liver tissue. In sum, our data show that UCB-MNC-sEV have no significant toxicity in vitro or in vivo, even when administered repeatedly at high concentrations, therefore confirming their safety profile and potential suitability for future clinical use.

## 1. Introduction

Extracellular vesicles (EV), secreted by most cell types, are described as key mediators of intercellular communication through the transport of a wide array of bioactive molecules such as proteins, RNA (including microRNAs), and DNA [1,2]. Being such a heterogeneous group, EV are catalogued into different subsets according to their cellular origin and size, ranging from the micron to the sub-micron dimension [3,4]. This diverse group of biological carriers participates in various physiological and pathophysiological processes, which sparked scientific interest in recent years. Aside from being useful diagnostic and prognostic tools, which can be obtained with minimally invasive techniques [5], EV are increasingly seen as potentially privileged drug carriers [6]. Their small size and lipid bilayer confer EV the ability to travel long distances in the body, including crossing the blood–brain barrier [7] without disturbing their cargo. At the same time, EV are easily internalized by cells and can have preferential targets [8]. Through exogenous drug loading, EV could specifically deliver therapeutic molecules to particular tissues or cells, minimizing systemic toxicity. Furthermore, unmodified EV have emerged as potential candidates for the replacement of cell therapies in different disease contexts [9,10]. In regenerative medicine, EV isolated from mesenchymal stromal cells (MSC) and mononuclear cells (MNC) have been demonstrated to successfully replace cell-based therapies, improving the function of damaged organs in animal models of ischemic diseases such as stroke [11,12], cardiovascular diseases [10,13,14], and chronic wounds [15,16].

As a promising new tool in regenerative medicine, several considerations must be taken into account before its clinical use. A major attribute of a new medicine before its commercialization relies on the establishment of its biological safety through appropriate toxicological studies [17]. In fact, the intravascular infusion of MSC has been documented to cause embolism and death in a mouse model [18], whereas MSC inoculated into pig infarcted myocardium were reported to induce adverse cellular growth such as cardiac sympathetic nerve sprouting [19]. For adverse effects such as these, it appears likely that the risk associated with EV administration, due to their small sizes, will be significantly lower or perhaps absent [20]. Moreover, EV are less prone to trigger immune responses and are unable to directly form tumours. A recent study by Zhu et al. reported minimal toxicity and immunogenicity of HEK293T-derived EV following repeated dosing in C57BL/6 mice [21]. Similarly, EV isolated from suspension human embryonic kidney Expi293F cells showed minimal toxicity and pro-inflammatory cytokine response following systemic administration into BALB/c mice [22]. However, the diversity of sources, isolation protocols, and manipulation of EV makes it difficult to transversally accept this claim for all EV-based therapies. Thus, it is increasingly urgent and fundamental to recommend standard techniques for the clinical-grade production and quality control of EV-based therapies as well as to define toxicology studies in order to accurately assess the safety of these new therapies.

In this study, EV isolated from human umbilical cord blood mononuclear cells (UCB-MNC) through a clinically transferable process were tested for their toxicity in vitro and in vivo. As described in a recent paper, the optimized methodology combines ultrafiltration and size exclusion chromatography (UF/SEC), yielding a small EV (sEV)-enriched product, with particle sizes ranging from 50 to 200 nm [23]. In comparison with ultracentrifugation, UF/SEC significantly reduces production time and improves process standardization [24,25] while maintaining sEV’s bioactivity [23]. This optimization in the manufacturing process may increase the confidence in UCB-MNC-sEV’s safety due to their strictly controlled production process. However, we cannot predict if these sEV induce metabolic or cell viability alterations, bring inflammatory and immune responses or hematologic variations.

UCB-MNC-sEV preparations consist of 80% sEV and 20% larger vesicles as well as proteins and lipids [23]. So far, the intravenous injection of similar EV showed no signs of toxicity [22]. The present study aimed to evaluate the cytotoxicity of UCB-MNC-sEV in vitro as well as determine their differential accumulation, distribution, and toxicity following repeated intravenous injection in a rodent model.

## 2. Material and Methods

### 2.1. UCB-MNC-sEV Isolation and Purification

Human UCB samples were obtained upon signed informed consent, in compliance with Portuguese legislation. The collection was approved by the ethical committee of Centro Hospitalar e Universitário de Coimbra, Portugal. Samples were stored and transported to the laboratory in sterile bags with an anticoagulant solution (citrate-phosphate-dextrose) and processed within 48 h after collection. UCB units were processed in an accredited cryobank (Crioestaminal, Cantanhede, Portugal) using an automated system AXP, according to the manufacturer’s recommendations.

After at least one week of storage, UCB-MNC were thawed and cultured at 2 million cells/mL in X-VIVO 15 serum-free cell-culture medium (Lonza, Basel, Switzerland), supplemented with 0.5 μg/mL of FMS-like tyrosine kinase-3 and 0.5 μg/mL of stem-cell factor, under ischemia (0.5% O_2_) conditions. Following 18 h of secretion, conditioned media were cleared by centrifugation and filtration, followed by ultrafiltration at 3 bar with a 100 kDa filter (Sartorius, Goettingen, Germany). Finally, the concentrated conditioned medium underwent size exclusion chromatography, and EV-enriched fractions were collected, concentrated, and stored at −80 °C until further use. A more detailed description of the EV purification process is published elsewhere (19).

### 2.2. UCB-MNC-sEV Characterization

Size distribution and concentration of UCB-MNC-sEV was measured with Nanosight LM (Malvern Instruments Ltd., Malvern UK), equipped with 638 nm laser and a CCD camera. The measurements were performed with a detection threshold set at 3, camera level set at 13, and a screen gain of 10. The blur and Max Jump Distance were set at 2. The sEV samples were diluted to obtain a number of particles per frame between 15 and 30. Readings were taken in 5 captures for 30 sec each, with a manual monitoring of temperature. A more detailed description of the EV characterization procedure and the results obtained is published elsewhere (19).

### 2.3. In Vitro Toxicity

To evaluate the possible cytotoxic effects of UCB-MNC-sEV in vitro, three UCB-MNC-sEV concentrations (1 × 10^10^, 5 × 10^10^, and 1 × 10^11^ particles/mL) were tested on different cell types. After 72 h of incubation, cell viability was evaluated through an XXT assay. PBS was used as vehicle control and all experiments were performed using an EV-depleted medium.

### 2.4. Cell Lines and Cell Culture Conditions

Normal human dermal fibroblasts (NHDF; PCS-201-012), human umbilical vein endothelial cells (HUVEC; PCS-100-010), and THP-1 cells (TIB-202; monocytes) were purchased from ATCC Cell Bank (ATCC, VA, USA). NHDF and HUVEC cell lines were cultured in T75 culture flasks, maintained in an incubator with controlled temperature (37 °C), 5% of CO_2_ level, and 90% humidity. A total of 60,000 cells were plated in a 96-well plate for the XTT assay. After 24 h of plating the cells and immediately before treatment, the culture medium was replaced by an EV-depleted medium (previously centrifuged for 14 h at 100,000× *g*).

THP-1 cells were cultured in T75 culture flasks, maintained in an incubator with controlled temperature (37 °C) and CO_2_ level (5%). These cells were grown to a density of 7 × 10^5^ cells/mL, plated in a 96-well plate in an EV-depleted medium, and rested in culture 24 h before treatment. To obtain macrophages, 100,000 THP-1 monocytes were seeded and incubated for 48 h with 25 nM PMA. Then, the PMA medium was replaced with a fresh medium and adherent cells were rested in culture for 24 additional hours. Immediately before treatment, the culture medium was replaced by an EV-depleted medium.

Human blood samples were obtained from Hospital Universitário de Coimbra, where donations were obtained from healthy volunteers after they provided their informed consent. Peripheral blood mononuclear cells (PBMC) were isolated by Lymphoprep gradient centrifugation (Stemcell Technologies, Vancouver, Canada) and plated on 96-well flat-bottom culture plates (Corning-Costar, Milan, Italy) at a density of 10^5^ cells/well.

### 2.5. Cell Viability-XTT Assay

To determine the viability of cells treated with UCB-MNC-sEV, an XTT assay (Applichem) was performed according to the supplier’s instructions. Briefly, the XTT mix was added to the medium and incubated at 37 °C for 3.5 h. The absorbance was read at 450 nm and 630 nm.

### 2.6. Animal Experiments

Animal testing protocols with Wistar Rats were approved on 17 December 2017 by the FMUC/CNBC ORBEA (Responsible Organism for Animal Welfare), with reference no. 147/20122017, and by the Portuguese National Authority for Animal Health (DGAV). All surgical and necropsy procedures were performed according to the applicable national regulations, respecting international animal welfare rules.

#### 2.6.1. In Vivo Biodistribution and Toxicity: 4 Weeks

Male Wistar Rats (12-week-old), purchased from Charles River and weighing between 250 and 400 g, were housed in a specific pathogen-free animal facility on a 12 h light/12 h dark regimen and fed a commercial diet (pellets) and acidified drinking water ad libitum.

To assess the biodistribution of systemically delivered UCB-MNC-sEV in rats, the vesicles were labelled with DiR (C18) dye (Thermo Fisher Scientific, MA, USA). Briefly, 50 μM of DiR dye was incubated with UCB-MNC-sEV or added to the vehicle (PBS) for 30 min at 37 °C. The samples were subsequently ultracentrifuged at 100,000× *g* for 2 h 18 min at 4 °C and filtered (0.2 µM). Before use, labelled-sEV were analysed by nanoparticle tracking analysis (NTA). After UCB-MNC-sEV modification with the DiR dye, 50 μL (5 × 10^10^ particles/mL) and the respective control were injected intravenously in rats tail vein twice a week. After four weeks of treatment, the fluorescent signal was observed with IVIS Lumina XR equipment (Caliper Life Sciences, Hopkinton, MA, USA). During the experiment, the animals’ weight and wellbeing were monitored. Signals of fighting, dehydration, excessive barbering, or malocclusion were closely monitored. After 4 weeks, the animals were euthanized through the recommended anaesthetic overdose of xylazine and ketamine. The most relevant organs, namely the liver, lungs, kidneys, spleen, pancreas, and heart were collected for further analysis of the fluorescence signal. Blood was also collected for further hemogram, leucogram, and biochemistry analysis. Urea, creatinine, alanine aminotransferase (ALT), aspartate aminotransferase (AST), and alkaline phosphatase (ALP) biochemical analyses were performed at the *Beatriz Godinho-Análises Clinicas* accredited laboratory. Several important haematology markers, including leukocytes, neutrophils, eosinophils, basophils, lymphocytes, monocytes, red blood cell count (RBC), haemoglobin, haematocrit, mean corpuscular volume (MCV), cell haemoglobin (CH), mean cell haemoglobin concentration (CHCM), and RBC distribution width (RDW) were selected for further toxicity assessment of UCB-MNC-sEV in vivo.

#### 2.6.2. In Vivo Toxicity: 6 and 12 Weeks

Male Wistar Han Rats (12-week-old), purchased from Charles River and weighing between 250 and 400 g, were housed in a specific pathogen-free animal facility on a 12 h light/12 h dark regimen and fed a commercial diet (pellets) and acidified drinking water *ad libitum*.

A total of 40 animals were randomized into 3 groups: Group 1—UCB-MNC-sEV 1 × 10^10^ particles/mL (1 × 10^9^ total particles) (14 animals); group 2—UCB-MNC-sEV 1 × 10^11^ particles/mL (1 × 10^10^ total particles) (14 animals); group 3—vehicle (12 animals). Each group was randomly sub-divided into 2 groups depending on the duration of treatment: 6 or 12 weeks. UCB-MNC-sEV (100 μL at 1 × 10^10^ or 1 × 10^11^ particles/mL) or control (vehicle) were intravenously injected into the tail vein, twice a week, over 6 or 12 weeks. The regimen of application was chosen based on the typical treatment schedule employed for the care of chronic wounds. During the experiment, the animals’ weight was monitored, and signals of fighting, dehydration, excessive barbering, or malocclusion were closely observed. After 6 or 12 weeks, the animals were euthanized and the organs and blood were collected, as described above.

#### 2.6.3. Histological Examination

Tissue biopsies were formalin-fixed in neutral buffered formalin, paraffin-embedded, cut into 4 μm sections, and stained with hematoxylin (Bio-Optica, Milan, Italy) and eosin (Thermo Fisher Scientific, MA, USA). Tissue sections were analysed in a Leica DM2000 microscope coupled with a Leica MC170 HD microscope camera (Leica Microsystems, Wetzlar, Germany) by a pathologist blinded to experimental groups.

### 2.7. Statistical Analysis

Data were analysed using GraphPad Prism 6 software. Statistical analysis was performed by ANOVA. The statistically significant level chosen was *p* value (*p*) < 0.05. Results were shown as mean ± standard error of the mean (SEM) and, when appropriated, they are marked with one asterisk (*) if *p* < 0.05, ** for *p* < 0.005, *** for *p* < 0.0005 and **** for *p* < 0.0001. Non-significant results (*p* > 0.05) are not stated.

## 3. Results

### 3.1. In Vitro Toxicity

**Figure 1:**
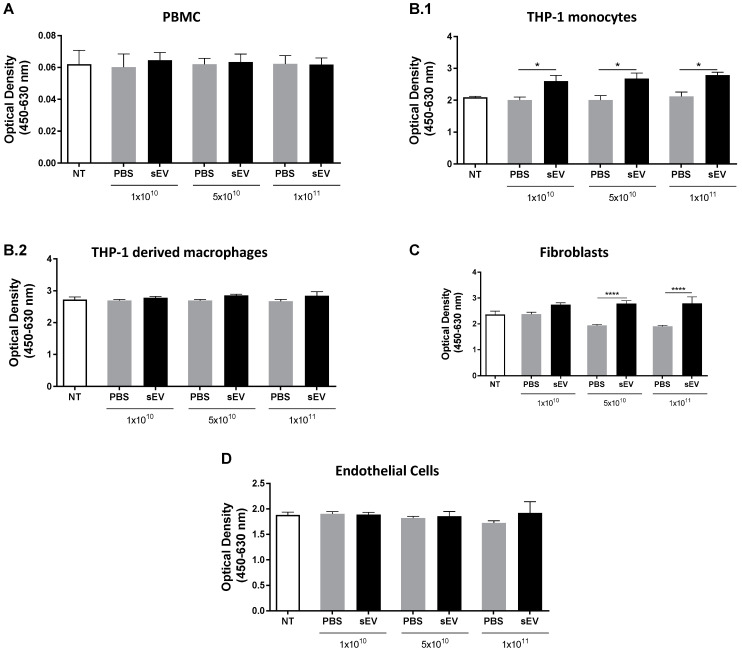
UCB-MNC-sEV do not induce cytotoxic effects in vitro. After cell seeding, cells received six doses of UCB-MNC-sEV, with the indicated particle concentration. After a 72 h treatment, cellular metabolic activity was measured by an XTT assay on (**A**) PBMC (n ≥ 3 per condition), (**B.1**) THP-1 monocytes (n ≥ 3 per condition), (**B.2**) THP-1 derived macrophages (n ≥ 3 per condition), (**C**) fibroblasts (n ≥ 6 per condition), and (**D**) endothelial cells (n ≥ 6 per condition). NT refers to “not treated” conditions. Results are presented as mean ± SEM. Statistical analyses were performed by ANOVA, * for *p* value < 0.05, and **** for *p* value < 0.0001. If not otherwise marked, no statistical difference was found between PBS- and sEV-treated cells.

The intravenous delivery of UCB-MNC-sEV represents both a potential administration route for future therapeutic applications, and a worst-case scenario of systemic exposure after local treatment. When injected intravenously, sEV first makes contact with a variety of cells, including circulating immune cells, endothelium, and connective tissue. Hence, we aimed to evaluate sEV cytotoxicity using primary cells and cell lines by challenging them with different particle concentrations. Three UCB-MNC-sEV concentrations (1 × 10^10^, 5 × 10^10^, and 1 × 10^11^ particles/mL) were tested on blood/immune system cells (monocytes and PBMCs), endothelial cells (HUVECs), and fibroblasts (NHDF). These concentrations were chosen based on previous efficacy tests, which demonstrated that 1 × 10^10^ particles/mL are therapeutically active (not shown). Therefore, concentrations that were 5- and 10-times higher were also included in this work. UCB-MNC-sEV were applied twice a day for 3 days. 72 h after treatment, cell viability was extrapolated from a metabolic assay (XTT). No evidence of cytotoxicity was found in any of the tested concentrations (Figure 1). More specifically, the metabolic activity of total PBMC, THP-1 monocytes and macrophages, NHDF, and HUVEC was not reduced when compared with control, indicating that no measurable cell death occurred during this timeframe. Worthy of note is the fact that there was a significant increase in the metabolic activity of sEV-treated THP-1 and NHDF as compared to vehicle controls. In the case of NHDF, this difference appears to have occurred due to an effect of the vehicle alone, which caused a reduction in the metabolic activity of control wells. By contrast, sEV administration significantly increased the absorbance of the THP-1 medium by about 31%, regardless of particle concentration. This increase could be the result of a boost in metabolic activity or of the higher proliferation index of sEV-treated cells, since XTT can be used as a proliferation readout. In line with the latter hypothesis, THP-1 cells induced to terminally differentiate by PMA showed no change in metabolic activity versus control wells, indicating that when the same cell line loses the capacity to proliferate, sEV administration no longer affects its metabolic activity. PBMC and HUVEC showed similar metabolic activity between sEV- and control-treated wells.

In sum, UCB-MNC-sEV do not elicit any cytotoxic effect in vitro when used between 1 × 10^10^ and 1 × 10^11^ particles/mL.

### 3.2. In Vivo Biodistribution and Toxicity: 4 Weeks

To confirm previous in vitro findings in an in vivo model, we first assessed the biodistribution and bioaccumulation of UCB-MNC-sEV. Wistar Han Rats were used and treated with dye-modified UCB-MNC-sEV in solution (5 × 10^10^ particles/mL) by tail vein injection, twice a week, over 4 weeks. The main organs were collected and analysed by IVIS.

**Figure 2:**
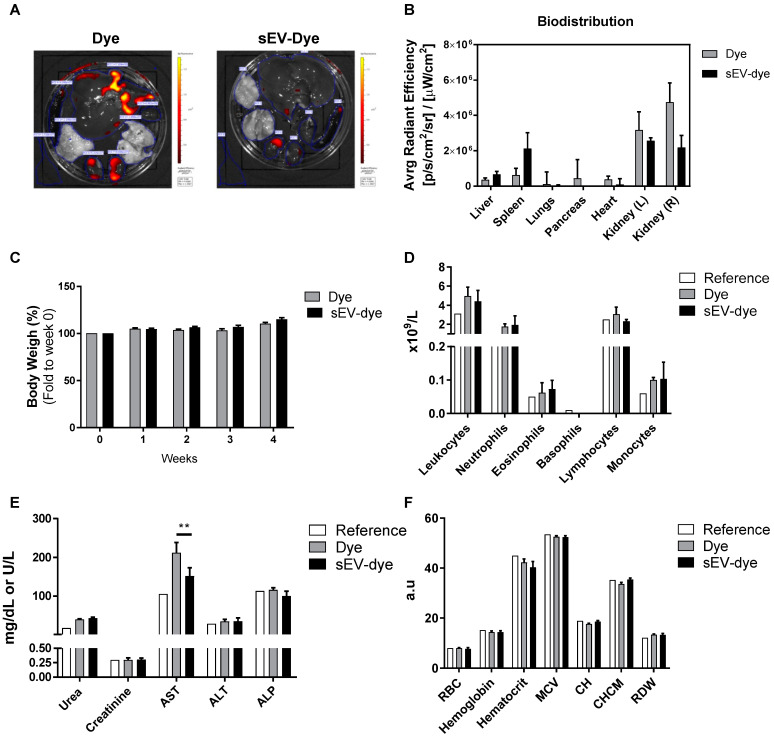
Biodistribution and toxicological profile of UCB-MNC-sEV in rats. Wistar Han rats were injected intravenously with fluorescently labelled UCB-MNC-sEV in PBS, twice a week for 4 weeks. After sacrifice, organs were analysed for accumulated fluorescence (IVIS) and blood was collected to evaluate signs of systemic toxicity. Control (Dye) animals were injected with the same dye concentration as used for labelled UCB-MNC-sEV. (**A**) Representative images of organs’ fluorescence. (**B**) Biodistribution of sEV-dye and dye alone. (**C**) Graphic representation of weight evolution during the 4-week experiment. Graphic representation of (**D**) leukogram, (**E**) biochemical, and (**F**) hemogram results acquired at the end of the experiment. Reference values were obtained from Charles River. Results are presented as mean ± SEM. Statistical analysis was performed by ANOVA (*n* = 4 rats per condition). If not otherwise marked, no statistically significant difference was found between dye and sEV-dye. RBC: red blood cell count; MCV: mean corpuscular volume (MCV); CH: cell haemoglobin; CHCM: mean cell haemoglobin concentration; RDW: RBC distribution width; a.u.: arbitrary units. ** *p* value < 0.01.

Data showed that after 4 weeks of treatment, dye-modified UCB-MNC-sEV and control were mostly accumulated in kidneys, followed by the spleen and the liver (Figure 2A,B). However, this biodistribution pattern cannot be attributed exclusively to sEV, as the respective control (dye without EV) shows a similar accumulation in the same organs, with no significant differences noted between the two groups.

As shown in Figure 2C,D, there was no significant impact on rats’ weight or circulating cell populations during the experiment, for either the sEV dye or the dye alone. Aspartate aminotransferase (AST), an enzyme indicative of liver damage when found in the circulation, was significantly higher in rats receiving the dye alone versus animals dosed with the sEV dye (Figure 2E). Given that this result was not accompanied by other markers of liver damage, it may not represent any significant tissue damage. Still, animals receiving the sEV dye showed normal levels of AST.

Finally, a classic hemogram analysis showed no relevant differences between reference values and the two test groups (Figure 2F).

### 3.3. In Vivo Toxicity: 6 and 12 Weeks

**Figure 3:**
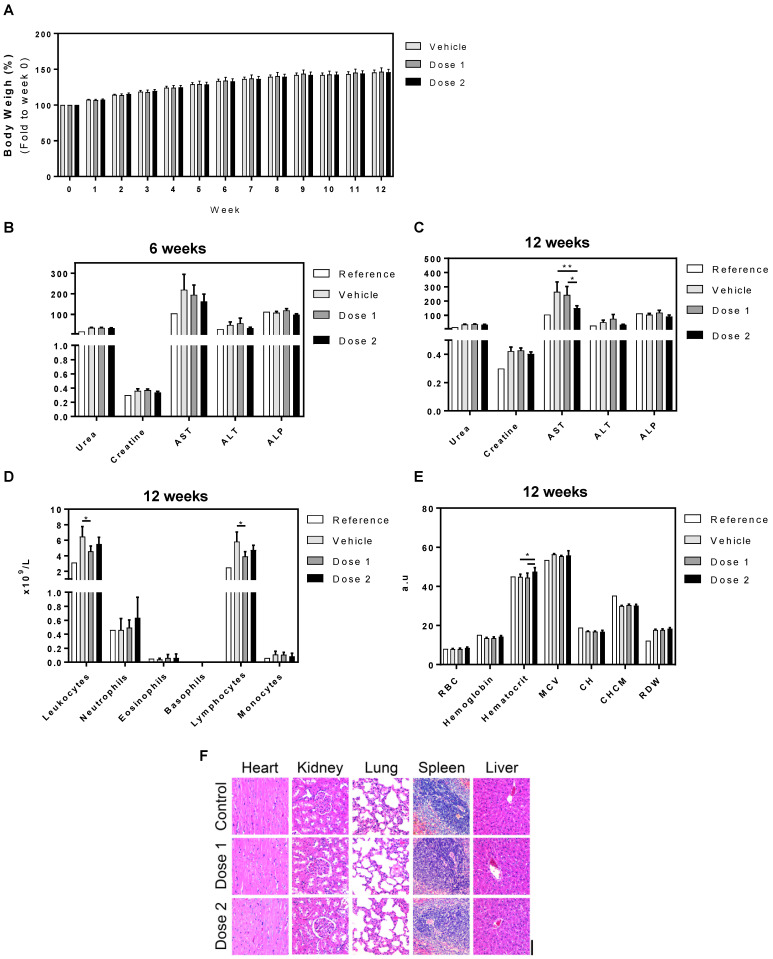
Toxicological profile of UCB-MNC-sEV in rats after repeated dose treatment (6 and 12 weeks). Wistar Han rats were injected intravenously with two UCB-MNC-sEV doses, twice a week for 6 or 12 weeks. Doses 1 and 2 refer to 1 × 10^10^ and 1 × 10^11^ particles/mL (1 × 10^9^ and 1 × 10^10^ total particles), respectively. After sacrifice, organs and blood were collected for analysis. Control (vehicle) animals were injected only with saline solution (PBS). (**A**) Graphic representation of weight evolution during the 12-week experiment. Graphic representation of (**B**,**C**) biochemical, (**D**) leukogram, and (**E**) hemogram results acquired at the end of the experiment. Reference values were obtained from Charles River. Results are presented as mean ± SEM. Statistical analysis was performed by ANOVA (*n* = 7 rats per condition). If not otherwise marked, no statistically significant differences were found between vehicle and dose 1 or dose 2. * for *p* value < 0.05, and ** for *p* value < 0.01 (**F**) Representative H&E microphotographs of heart, kidney, lung, spleen, and liver from untreated and treated rats exposed to two different doses. Original magnification 20× (bar, 100 µm).

We next aimed to verify the bioaccumulation effects and toxicity of UCB-MNC-sEV in a worst-case scenario study (high dose, intravenous route, and repeated long-term administration). For that purpose, Wistar Han rats received UCB-MNC-sEV in a saline solution (1 × 10^10^ and 1 × 10^11^ particles/mL, respectively, 1 × 10^9^ and 1 × 10^10^ total particles) by tail vein injection, twice a week, over 6 or 12 weeks. The blood and main organs were collected and analysed. As shown in Figure 3A, UCB-MNC-sEV have no significant impact on rats’ weight over time. All animals showed an increase in body weight, suggestive of good general health and appropriate access to food and water.

Blood biochemistry after 6 weeks showed a trend toward the reduction of AST with the highest sEV dose, which became significant after 12 weeks in comparison with vehicle and lower dose sEV. Additionally, total leukocytes and lymphocytes were slightly reduced with both sEV doses, while erythrocyte volume was slightly increased. Despite these slight differences between treatment groups, all parameters are in line with reference values and are likely of no particular relevance. Finally, we conducted histological analyses of major functional organs, including heart, kidney, lung, spleen, and liver, and observed no morphological changes or signs of toxicity in any of the organs analysed (Figure 3F).

## 4. Discussion and Conclusions

In this study, the toxicology of UCB-MNC-sEV was analysed to assess the vesicles’ feasibility as therapeutic agents and to predict potential adverse effects. EV contain most of the desirable features of an ideal drug delivery system such as the intrinsic ability to target tissues, biocompatibility, and presumable minimal toxicity [26]. Nevertheless, it is imperative to know the toxicological risks of sEV before their clinical use [17].

Collectively, the results obtained provide evidence for the absence of significant toxicity after treatment with UCB-MNC-sEV. In vitro, the results show no UCB-MNC-sEV cytotoxicity since there is no significant decrease in relative cell number or cell metabolic activity, as measured with an XTT assay. Notably, dermal fibroblasts and monocytic cells presented a significant increase in metabolic activity, presumably due to a direct effect of sEV on cell survival/proliferation. While a boost in metabolism could also point toward an increase in cellular secretory activity, the fact that terminally differentiated cells (THP-1-derived macrophages) do not behave similarly indicates that proliferation is a more likely scenario. These outcomes are in line with previous UCB-MNC-sEV effects observed in wound healing [16,23], and with reports from other EV sources [27,28,29]. Overall, our in vitro data demonstrate that UCB-MNC-sEV do not significantly decrease the cellular metabolic activity of different cell types, at least within the range of concentrations tested, until after 72 h of contact with sEV.

Biodistribution and bioaccumulation studies, after 4 weeks of treatment, revealed no significant differences in weight, hemogram, and leukogram between the evaluated groups. The distribution and accumulation of systemically-administered UCB-MNC-sEV was measured using DiR-labelled vesicles, as reported by other authors [30,31,32]. Dye-modified sEV seem to accumulate in the liver and spleen, a pattern consistent with previous reports examining EV biodistribution in mice [30,31]. Nevertheless, these reports demonstrated an inverse order of fluorescence accumulation, being higher in the liver and followed by the spleen, lung, and brain [33]. Therefore, we believe in a preferential accumulation in spleen compatible with sEV’s immunomodulatory mode of action. Hepatic and splenic bioaccumulation are related to the uptake by resident phagocytes in the liver, as well as macrophages and B cells in the spleen, which are part of the mononuclear phagocytic system (MPS), or of the reticuloendothelial system (RES), as described in other biodistribution reports [30,34]. In addition, much of the signal detected by IVIS in both treated and control groups were noticed in the kidneys. In view of the substantial variability and the absence of statistically significant data, consideration should be given to the hypothesis that the data obtained may have been influenced by the dye excretion pathway, and that the obtained results do not represent the bioaccumulation pattern of UCB-MNC-sEV. Lipophilic dyes such as DiR can uncouple from EV membranes and incorporate other cell membranes, or the mere presence of non-covalent or covalent dyes might alter the interaction between sEV and target cells, therefore affecting sEV internalization and biodistribution. Nevertheless, fluorescent labelling is virtually the only available strategy able to detect naturally-produced, unmodified EV and has been successfully used in the literature.

Biochemistry analyses revealed that DiR alone seems to induce a slight hepatoxicity, as seen by the increased AST levels. Although this result likely bears little toxicological relevance, it is worth noting that when the dye is incorporated in sEV, AST levels were significantly decreased as compared with dye alone. These results suggest either an sEV hepatic protective mechanism or the dye’s inability to interact with membranes once it is already conjugated with sEV. The remaining blood analyses, including leukogram and hemogram, showed no difference between dye alone and dye-sEV, confirming that neither test component caused measurable toxicity after systemic administration for 4 weeks.

Similarly, the 6- and 12-week-long repeated administration of UCB-MNC-sEV at 1 × 10^9^ or 1 × 10^10^ total particles/dose had no major effect on general markers of toxicity. Overall, rats receiving vehicle showed a significantly higher level of circulating AST, leukocytes, and lymphocytes in comparison with sEV-dosed animals. Despite the statistically significant differences between vehicle and sEV administrations, these generally pointed towards a normalization closer to reference values after sEV administration. While we cannot offer a definitive explanation for the shifts observed between reference and vehicle, this phenomenon could perhaps be related to a slight inflammatory reaction due to the repetitive IV infusions. If that is the case, sEV counter the inflammatory response and restore laboratory values to normal. A recent report from Driedonks et al. [35] raised the possibility that repeated administrations of Expi293-derived EV to macaques could be immunogenic to a degree, as measured by the faster decay of EV in circulation. In our studies, we did not find any evidence of UCB-MNC-sEV immunogenicity. However, it should be noted that the experiments described in this work were not designed to measure immunogenicity, a question that still remains to be addressed before the clinical application of sEV. Finally, the histological analyses corroborate the absence of toxicity induced by UCB-MNC-sEV administration, since all sections analysed depict normal histological features. Similarly, the administration of Expi293F-derived EV to mice did not result in any histopathological changes or increases of liver transaminases, supporting minimal or absent liver damage [22].

To conclude, no harmful effects were caused by the intravenous injection of the highest tested dose of UCB-MNC-sEV, which is 40 times higher than the predicted therapeutic dose [16,23]. As this experiment was designed to resemble a worst-case scenario toxicity study, we can conclude that UCB-MNC-sEV use is safe at concentrations of up to 1 × 10^11^ particles/mL (or 1 × 10^10^ total particles) when administered intravenously twice a week for three months. By confirming their safety in a rodent model, these results support the clinical development of UCB-MNC-sEV.

## Data Availability

Data presented in this study can be made available upon request from the corresponding author.
